# Hepatitis B virus infection among sexually active individuals in Nigeria: a cross-sectional study

**DOI:** 10.11604/pamj.2018.30.155.14886

**Published:** 2018-06-20

**Authors:** Yewande Nejo, Adedayo Omotayo Faneye, Babatunde Olusola, Solomon Bakarey, Adebowale Olayinka, Babatunde Motayo, PERVI Study Group

**Affiliations:** 1 Department of Biological Sciences, Bowen University Iwo, Nigeria; 2 Department of Virology, College of Medicine University of Ibadan, Nigeria; 3 Institute for Advanced Medical Research and Training, University of Ibadan, Nigeria

**Keywords:** Sexual transmission, chronic HBV infection, Hepatitis B virus nucleic acid related antigen, male gender, STI clinic attendees, occult infection, HBV life cycle

## Abstract

**Introduction:**

Hepatitis B virus (HBV) infection is a major health challenge in sub-Saharan African countries. Chronic HBV infection is a risk factor for severe disease progression. Perinatal and sexual transmissions of Hepatitis B virus are the main routes of infection in HBV endemic countries like Nigeria. However, there is paucity of data as regards the major contributory route of transmission to chronic HBV infection in this region. Also, in Nigeria, not everyone at high risk of the infection has been identified. Therefore our study investigated the prevalence of HBV infection among sexually active individuals in Nigeria.

**Methods:**

Blood samples collected from 463 participants (360 sexually active individuals and 103 teenagers) recruited from health institutions across the country were tested for the presence of HBsAg, and HBV nucleic acid related antigen (HBVNRAg) by ELISA. Positive samples were further tested for the presence of HBeAg and antiHBe by ELISA. Data were analyzed using Chi-square and binary logistic regression at p = 0.05.

**Results:**

HBsAg and HBVNRAg were detected in 10.4% and 7.6% of the participants respectively. STI clinic attendees had the highest prevalence for HBsAg (17%; p = 0.002). Teenagers had the lowest HBsAg (1.9; p = 0.002) and HBVNRAg (2.9%; p = 0.0001) prevalence rates. Male gender (p = 0.01) and reproductive age group (p=0.009) were the major predictors of chronic HBV infection.

**Conclusion:**

Sexual transmission was identified as the major contributor to chronic HBV infection. Sexually active individuals especially those with STIs are high risk groups for chronic HBV infection. Interventions targeted at this group is therefore recommended.

## Introduction

Hepatitis B virus infection is one of acute to chronic illnesses, the major blood transmissible infections known to man with about 400 million people chronically infected globally [1, 2]. HBV prevalence is highest in sub- Saharan Africa and East Asia, where 5-20% of the adult population is infected [3]. In Africa up to 15 to 60% of the general population are positive for at least one of the serological markers of HBV infection. HBV causes a spectrum of ranging from inactive chronic carrier status to progressive chronic hepatitis that leads to end-stage cirrhosis and primary liver cancer [4]. Chronic HBV infection is defined by the prevalence of HBsAg carriage and it is a risk factor for disease progression in infected individuals [5]. Countries with HBsAg prevalence above 8% are said to be HBV endemic [6]. HBV infection is endemic in Nigeria and is an important cause of morbidity and mortality despite availability of vaccines in the country [7].

Hepatitis B virus belongs to the virus family *hepadnaviridae* with a double-stranded circular DNA genome [8]. The virus has proteins that are important markers for diagnosis and management. These proteins are hepatitis B surface antigen (HBs Ag); the outer protein coat of the virus, Hepatitis B envelope antigen (HBeAg), which is shed when the virus is replicating and hepatitis B core antigen (HBcAg) which is abundant in the hepatocytes. Chronic HBV infection is defined by the prevalence of HBsAg which is used to group regions and countries to low, medium and high HBV endemic areas [6]. The life cycle of HBV infection in human hosts can be divided into four stages [9]. The first two stages are grouped as the replicative phase while the last two as the integrative phase. In the first stage, HBsAg, HBV DNA and HBeAg are detected in blood while Anti HBe is undetectable, that is, negative. This active period is defined by immune tolerance in which there is ongoing active viral replication characterized by high levels of HBV DNA and host infectiousness. The second stage is similar to the first except that there is a reduced HBV DNA level and an elevated liver enzymes profile. During the third stage, HBV DNA is negative, HBsAg and AntiHBe is detectable in blood while HBeAg is, negative. At this time, active viral replication ends and host immune control is achieved. There is complete clearance of the virus in the fourth stage with HBsAg becoming undetectable and host infectiousness drastically reduced [9].

Occult infection has been defined as the presence of replication-competent HBV DNA in the liver but without detectable HBsAg in the serum. [10] Also, in developing countries, studies have used HBV nucleic acid related antigen (HBVNRAg) as a surrogate marker for HBV DNA detection and quantitation due to the high cost of the test [11-13]. Table 1 gives a description of the serological markers detected at each stage of the HBV life cycle in humans. Sub-Saharan Africa has the highest endemicity for chronic HBV infection with varying prevalence of the infection occurring within diverse groups exposed through occupational hazard or behavior [5,6]. In countries in this region, weak public health infrastructures and poor surveillance systems are critical factors hampering the control of HBV infection [14, 15]. Also, high risk groups are not properly identified in these areas [16]. It is important to determine high-risk groups as they have been shown to be the determinants of HBV prevalence and incidence globally [17]. Therefore, to achieve HBV control, it is pertinent that high risk groups are properly identified and monitored. However, studies have focused on the perinatal and percutaneous routes of HBV transmission in identifying high risk groups for HBV infection in these countries [15].

**Table 1:** Serological markers detected at each stage of HBV life cycle in human hosts

	Active Replication Phase			Integrative Phase		Occult
Marker	Acute	Stage 1	Stage 2	Stage 3	Stage 4	
*HBsAg*	+ve	+ve	+ve	+ve	-ve	-ve
*HBVNRAg*	+ve	+ve	+ve	-ve	-ve	+ve
*HBeAg*	-ve	+ve	+ve	-ve	-ve	-ve
*Anti Hbe*	-ve	-ve	+ve	+ve	+ve	-ve

Perinatal transmission has been said to be the major route of HBV transmission in areas of high endemicity, although scanty data support this claim in sub-Saharan Africa [18]. Another known route of HBV transmission is the sexual route [19, 20]. However, the contributory role played by these modes of transmission in sustaining a very high burden of chronic HBV infection in this region has not been fully described. This study was therefore designed to investigate the prevalence of HBV infection in Nigeria among groups of sexually active individuals : pregnant women, young teenagers, adults and patients attending STI clinics. The burden of HBsAg carriage, host infectiousness and immune control among these groups were also determined.

## Methods

### Study design

The study was cross sectional in approach, HBV serological markers (HBsAg and HBVNRAg) were tested using commercially available ELISA test kits. Those that were screened positive for either one or both markers were then further tested for HBeAg and Anti HBe.

### Study population

A total of 463 individuals within the age range of 15 to 60 years attending either Sexually Transmitted infection (STI) or antenatal clinics as well as those presenting at medical outpatients units of Bowen University Teaching Hospital, Ogbomosho (n = 92), Oyo state, Bowen University Hospital, Iwo (n = 92), Osun state, Federal Medical Centre, Ido-Ekiti (n = 94), Ekiti state, Primary Health Centre, Ogijo (n = 92), Shagamu, Ogun State and Epidemiological Unit of the Ministry of Health, Jos (n = 93), Plateau State who consented to participate in the study were enrolled between January 2014 and December 2015. Ethical approval for this research was obtained for this study. Procedures were followed in accordance with the ethical standards of the committee and with the Helsinki Declaration of 1975, revised 2000. Written informed consent was obtained from each patient before participating in the study. Inclusion criteria: Sexually active individuals aged 15 to 60 years attending aforementioned clinics that consented to participate in the study were randomly selected for this study. Exclusion criteria: None consenting and critically ill individuals were excluded.

### Ethical consideration

This research was approved by Health Ethical Research Committee, Kwara State Ministry of Health, Ilorin, Kwara State, Nigeria with approval number MOH/KS/EC/777/103.

**The protocol for this study conformed to the ethical guide lines of 1975 declaration of HelsinkiConfidentiality:** All information collected from the participants was carefully safeguarded and only made available to authorized personnel.

**Beneficence to subjects:** Participants in this study were tested free for HBV and the result made available to the participant and their doctors to aid treatment.

**Non- maleficence to subjects:** This research was carried out without any intention of bringing harm to the participants. The rights, integrity, and safety of all participants were protected. Also, the samples were collected with minimal discomfort to the participants as much as possible.

**Voluntariness:** Participation in this research is voluntary, and only after the participant has read, and understood the consent document and consent to participate in the study. Each participant was informed of his or his or her freedom to decline from participation at any point. Assents were obtained from parents or guardians of participants less than 18 years recruited for this study.

### Sample collection

Five milliliters of blood was collected by venipuncture from each participant into anticoagulant (EDTA) bottles after giving their informed consent. The plasma samples were separated from the blood by centrifuging at 3000rpm for 10 minutes and pipetted into labeled plain bottles which were stored at -20oC until assay was done.

### Testing for HBV markers

Plasma samples were allowed to thaw to attain room temperature before analysis. The serological testing for markers of HBV infection, HBsAg, HBeAg/ anti-HBeAg antibody, HBVNRAg was done using ELISA kits by Wantai (Beijing wantai Biological Pharmacy Enterprise, Beijing, China) according to the manufacturer’s instruction. All the samples were tested for HBsAg and HBVNRAg. Positive samples for either HBsAg or HBVNRAg were further tested for HBeAg and anti-HBe. The optical density of the plates was read using the Emax Endpoint Microplate Reader (Molecular devices, California, USA). Controls were read first before test samples and results were calculated based on cut off values, set as mean value of 3 negative controls + 0.12 O.D according to the manufacturer’s instruction. Samples with equivocal results were retested and if afterwards remains equivocal were regarded as negative.

### Operational definition of HBV infection stages for study participants

HBsAg, HBVNRAg, HBeAg and AntiHBe seropositivities were used to define the various stages of HBV infection of our study participants as shown in Table 1. Acute HBV infection is defined by detections of HBsAg and HBVNRAg only. In stages 1 and 2 of HBV infection; HBsAg, HBVNRAg and HBeAg are positive. Only difference being that AntiHBe is also positive in stage 2. For stage 3, only HBsAg and AntiHBe are positive [9]. Occult HBV infection is defined by the seropositivity of only HBVNRAg [10].

### Statistical analysis

Data were analyzed by Statistical Package for the Social Sciences (SPSS) version 21. Means and standard deviations were used to describe variables such as age, sex and category. Categorical variables were analyzed using non-parametric tests such as chi square test and binary logistic regression. Indicator of statistical significance was set at P<0.05 at 95% confidence interval.

## Results

### Characteristics of the study population

**Figure 1:**
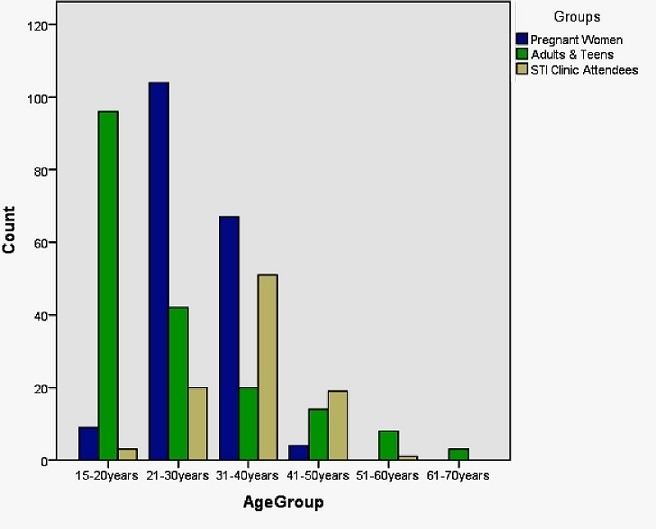
Age and Gender distribution of study participants

**Figure 2:**
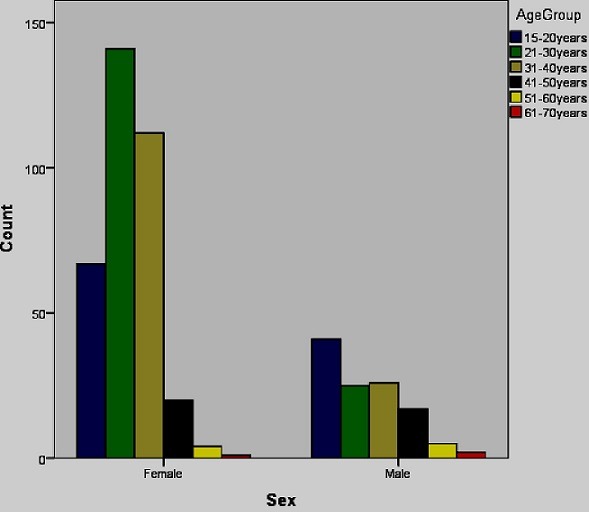
Age group distribution of participants per stratified groups

Four hundred and sixty three persons were recruited into this study. Three hundred and forty six (74.7%) of these individuals were female and the mean age ± SD of the participants were 34 ±2 years. Figure 1 and Figure 2 show a summary of the distribution of the study groups.

### Overall distribution of HBV markers

Of the 463 participants screened, 48 (10.4%) had HBsAg while 35 (7.6%) had HBVNRAg as shown in Table 2. A total 54 persons tested positive for HBsAg and/or HBVNRAg. Twenty one of the 54 persons had only HBsAg while eight had only HBNRAg; 27 had both. Out of the 54 samples tested for HBeAg/Ab, 11 had HBeAg while 34 had anti-HBe (HBeAb). Three samples had all the biomarkers tested (HBsAg, HBNRAg, HBeAg and anti-HBe). Overall distribution of the markers by age and gender is described in Table 3. Group specific prevalence for HBsAg revealed that age group 15-20 years had the lowest rate (3.6%) while age group 31-40 years had the highest rate (14.5%) (p = 0.043). Males (11.1%) had higher HBsAg prevalence than females (6.4%) (p = 0.016). Binary logistic regression analysis of study variables for HBsAg showed that persons in age group 31-40 years were over five times more at risk of HBV infection than other age groups in the study (P = 0.009). Males had an odds ratio of 2.849 (p = 0.01) as against their female counterparts (See Table 4).

**Table 2:** HBsAg and HBVNRAg seroprevalence rates in the study population

Category	HBsAg	P Value	HBVNRAg	P Value
	No Positive (%)		No Positive (%)	
Pregnant women (n=184)	17(9.2)		13(7.1)	
Adults (n=82)	13(15.9)		15(18.3)	
Teens (n=103)	2(1.9)		3(2.9)	
STI Clinic attendees (n=94)	16(17.0)	0.002	4(4.3)	0.0001
Total (n=463)	48(10.4)		35(7.6)	

The differences in HBsAg (P = 0.002) and HBVNRAg prevalences (P=0.0001) by category reached significant levels

**Table 3:** Age and gender distribution of HBsAg positivity in the study population

Variable		P-Value
**Gender**	**HBsAg pos (%)**	
Male (n=117)	19 (16.2)	
Female (n=346)	29 (8.4)	0.016*
**Age-group**		
15-20 (n=110)	4 (3.6)	
21-30 (n=116)	20 (12)	
31-40 (n=138)	20 (14)	
41-60 (n=37)	4 (10.8)	0.043*

Differences in HBsAg positivity by gender and Age-group reached significant levels

**Table 4:** Binary rewgression analysis of age and gender in relation to HBsAg positivity

Variable	Prevalence	Odds Ratio	P-value (95% CI)
**Age**			
15-20	3.60%	1	
21-30	12%	4.878	0.009* (0.064-0.705)
31-40	14%	5.227	0.009* (0.058-0.700)
>40	10.80%	2.578	0.229 (0.124-2.493)
Gender			
Male	16.20%	2.849	0.01* (1.275-6.311)
Female	8.40%	1	
Category			
Pregnant Women	9.20%	1.03	
Adults & Teens	8.10%		
STI Clinic Attendees	17%	1.365	0.05* (0.238-1.034)

The highest predictors of HBsAg by Age, Gender and Category are 31-40 years, Male gender and STI Clinic Attendees respectively. 1: reference group

### Distribution of HBV markers among the study groups

Twenty (10.8%), 16 (17.0%) and 18 (9.7%) of pregnant women, STI clinic attendees and adults and teenagers tested were positive for HBsAg and/or HBVNRAg respectively. Distribution of HBV infection markers among the study groups is described in Table 2. STI clinic attendees had a significantly higher prevalence of HBsAg carriage (17%) compared to other groups. Overall, STI clinic attendees had the lowest prevalence of acute and active HBV replication (p = 0.211; p = 0.118 respectively). This group however had the highest burden of immunologically controlled HBV infection (p = 0.006) across the groups (see Table 5).

**Table 5:** Showing categorical prevalence rates of HBV markers of infection

	Participants				
HBV Infection	Pregnant Women (%)	Adults & Teens (%)	STI Clinic Attendees (%)	Total	P value
*Acute Infection*	4(44.4)	4(44.4)	1(11.1)	9	0.211
*Stage 1*	0(0)	0(0)	2(100)	2	0.118
*Stage 2*	1(33.3)	2 (66.6)	0 (0)	3	0.118
*Stage 3*	2 (18.1)	0(0)	9 (81.8)	11	*0.006*
*Occult Infection*	4(80)	1(20)	0(0)	5	0.406

## Discussion

Nigeria like many other sub Saharan African countries has a high burden of chronic HBV infection with over 14% of its population exposed to the infection between 2000 and 2013 [7]. Despite this, the country’s health care program only has viral hepatitis prevention and control strategy for a few high risk groups such as health-care workers, health-care waste handlers and blood transfusion recipients. This study evaluated the prevalence of HBV infection among adults, adolescents and pregnant women in Nigeria. Our report of 10.4% HBsAg prevalence is higher than a recent report of 9.3% among blood donors in Abeokuta, Nigeria [21]; also higher than the report from South- South, Nigeria [22]. Furthermore, findings from Ejele et al., (2005) [23] and Olokoba et al., (2009) [24] in the country recorded far lower prevalence rates, of 1.1% and 2.4% respectively. This variation could be as a result of the study populations as studies have shown varying HBV prevalence among diverse groups and regions in the country and in fact across sub Saharan Africa [5, 6, 25]. The type of test kit used in screening may also be considered although this bias is inherent for all studies [25].

Gender associated HBsAg prevalence showed that males are 2.8 times more likely to get infected with HBV compared to their female counterparts. This variation has been previously reported [19, 26] and the authors noted that behavioral differences between the male and female gender may account for this difference. However, an altered pattern of liver apolipoprotein A-I isoforms in males may also be a plausible biological explanation [27]. Studies have shown that males are less likely to clear HBV infection and at a higher risk of progressing to chronic infection [25, 28].

The prevalence of HBV infection among different groups in our study population suggests that pregnant women, adults and STI clinic attendees have higher rates than teens (Table 1). Findings from this study also show that persons in the reproductive age group (21-40 years) are significantly at a higher risk of HBV infection when compared to teenagers and the elderly (Table 4). Heterosexual transmission seems to be a significant mode of HBV transmission in our study setting, moreso that our study found a significantly higher prevalence of chronic HBV among STI clinic attendees across the study groups. This is contrary to previous hypothesis that majority of HBV infections, particularly chronic infections were most likely acquired prenatally and during infancy [29]. This should translate to teenagers having a higher prevalence of chronic HBV infection than older sexually active persons. However, our observation is in the contrary to this. Recent studies seem to support the view that vertical transmission is not a major contributory factor to chronic HBV infection in highly endemic areas [30,31]. Although teenagers that participated in the study could also have been infected through sexual means, it may be very difficult to verify this claim. This in our opinion is modified by sexually active adults that may also have been infected by the perinatal route and not necessarily during sexual exposure.

Prevalence of HBsAg carriage, host infectiousness and immunologically controlled HBV infection varied across the group (see Table 5). As stated earlier, HBsAg status was significantly associated with sexual transmission. However, there was an increase in HBV prevalence among STI clinic attendees and the general population as the infection moved from acute to the chronic and /or immunologically controlled phase. A reverse trend was however observed among pregnant women although these differences did not reach significant levels because of the small sample size. HBV infected pregnant women and younger people are likely to be more infectious [25] than persons on treatment for sexually transmitted infections although this group at a higher risk of chronic HBV infection. This pattern strongly favors the view that perinatal and vertical transmission does not contribute significantly to high levels of chronic HBV infection in endemic countries. Cases of occult HBV were also observed in this study as five samples tested positive for HBVNRAg and negative for HBsAg. Interestingly, four of the people with occult HBV infections were pregnant women (Table 5). Pregnancy leads to a state of immune suppression which may enable the development of an occult infection, although this is yet to be verified [32]. This hypothesis however opens up a research gap on the relationship between pregnancy state and occult infection in Nigeria. We note the difficulty in ascertaining unprotected sexual relationships among the teenagers that participated in the study.

## Conclusion

The findings from this study have further highlighted the sustained transmission of HBV among Nigerians; male gender and reproductive age group (21-40 years) were identified as major contributors to HBsAg carriage and chronic HBV infection in Nigeria. This study also shows that acute/active HBV replication declines with increasing age and sexual exposure. Sexually active individuals and persons with STIs are at high risk of chronic HBV infection. Interventions directed at these groups of people will help in the control of the spread of the virus.

### What is known about this topic

- Nigeria is hyper endemic with chronic HBV infection. Chronic HBV infection is a major determinant of severe disease progression. Perinatal and sexual modes of HBV transmission are considered major routes of transmission of the virus in the country.

### What this study adds

- Sexual mode of transmission is a major contributor to chronic HBV infection in Nigeria;
- STI clinic attendees are high risk groups for HBV infection in Nigeria;
- HBV host infectiousness declines with increasing age in the country.

## Competing interests

The authors declare no competing interests.
